# Epidemiology of *Pneumocystis jirovecii* Pneumonia and (Non-)use of Prophylaxis

**DOI:** 10.3389/fcimb.2020.00224

**Published:** 2020-05-15

**Authors:** Albert Dunbar, Alexander Schauwvlieghe, Sheruna Algoe, Jaap J. van Hellemond, Marijke Reynders, Stefaan Vandecasteele, Jerina Boelens, Pieter Depuydt, Bart Rijnders

**Affiliations:** ^1^Department of Internal Medicine, Infectious Diseases, Erasmus University Medical Center, Rotterdam, Netherlands; ^2^Department of Hematology, Erasmus MC Cancer Institute, Rotterdam, Netherlands; ^3^Department of Hematology, Ghent University Hospital, Ghent, Belgium; ^4^Department of Medical Microbiology & Infectious Diseases, Erasmus University Medical Center, Rotterdam, Netherlands; ^5^Department of Laboratory Medicine, Medical Microbiology, AZ St-Jan Brugge-Oostende Hospital, Bruges, Belgium; ^6^Departments of Nephrology and Infectious Diseases, AZ St-Jan Brugge-Oostende Hospital, Bruges, Belgium; ^7^Department of Laboratory Medicine, Ghent University Hospital, Ghent, Belgium; ^8^Department of Intensive Care Medicine, Ghent University Hospital, Ghent, Belgium

**Keywords:** *Pneumocystic jirovecii* pneumonia, *Pneumocystis jiroveci* (carinii) pneumonia, prophylaxis, Trimetoprim- sulfamethoxazole, immunocompromidsed patients

## Abstract

**Objectives:** *Pneumocystis jirovecii* pneumonia (PCP) is an AIDS-defining illness. In patients with HIV, the benefit of PCP prophylaxis is well-defined when the CD4 T-cell count decreases below 200 cells/μL. In other immunocompromised patients, the value of PCP prophylaxis is not always as well-established. This study aimed to describe the epidemiology of PCP in recent years and assess how many patients with PCP did or did not receive prophylaxis in the month preceding the infection.

**Material and Methods:** A multicenter retrospective study was performed in 3 tertiary care hospital. A list of patients that underwent broncho-alveolar lavage sampling and *Pneumocystis jirovecii* (PJ) PCR testing was retrieved from the microbiology laboratories. An in-house PJ quantitative PCR (qPCR) was used in each center. A cycle threshold (Ct) value of ≤ 28.5–30 was considered a probable PCP. For patients with a positive PJ qPCR but above this threshold, a predefined case definition of possible PCP was defined as a qPCR Ct value ≤ 34–35 and both of the following criteria: 1. Clinical and radiological features compatible with PCP and 2. The patient died or received PCP therapy and survived. Patient files from those with a qPCR Ct value ≤ 35 were reviewed to determine whether the patient fulfilled the case definition and if PCP prophylaxis had been used in the weeks preceding the PCP. Disease-specific guidelines, as well as hospital-wide guidelines, were used to evaluate if prophylaxis could be considered indicated.

**Results:** From 2012 to 2018, 482 BAL samples were tested. Two hundred and four had a qPCR Ct value ≤ 35 and were further evaluated: 90 fulfilled the definition of probable and 63 of possible PCP while the remaining 51 were considered colonized. Seventy-four percentages of the patients with PCP were HIV-negative. Only 11 (7%) of the 153 patients had received prophylaxis, despite that in 133 (87%) cases prophylaxis was indicated according to guidelines.

**Conclusion:** In regions where HIV testing and treatment is available without restrictions, PCP is mainly diagnosed in non-HIV immunocompromised patients. More than four out of five patients with PCP had not received prophylaxis. Strategies to improve awareness of antimicrobial prophylaxis guidelines in immunocompromised patients are urgently needed.

## Introduction

*Pneumocystis jirovecii* (PJ), formerly known as *Pneumocystis carinii* species, is a unicellular eukaryotic and ubiquitous yeast-like fungus and is the cause of *Pneumocystis jirovecii* pneumonia (PCP), a life-threatening opportunistic disease (Stern et al., 2014). It is a well-established complication in HIV-positive patients but with the advent of combined antiretroviral therapy (cART) and the use of prophylaxis in HIV-positive patients, the epidemiology shifted to non-HIV immunocompromised hosts in countries where HIV testing and access to cART is in place. The non-HIV infected immunocompromised host encompasses patients after a solid organ transplant (SOT), allogeneic hematopoietic stem cell transplant recipients, patients receiving immunosuppressive therapies for autoimmune and inflammatory conditions, with genetic primary immune deficiency disorders and to a lesser extent those treated for solid malignancies (White et al., 2017). The clinical course of PCP is more severe in non-HIV patients with increased length of hospital stay, higher rates of mechanical ventilation and intensive care unit (ICU) admission compared with HIV-infected patients (Roux et al., 2014; Sokulska et al., 2015). Furthermore, a significant discrepancy in mortality rates from PCP exists between HIV-infected patients and non-HIV infected immunocompromised patients, 10–20 vs. 30–60%, respectively (Morris and Norris, 2012; Stern et al., 2014; Kotani et al., 2017).

The role of PCP prophylaxis in the HIV-negative immunocompromised host is not well-established. A Cochrane meta-analysis assessed the effectiveness of PCP prophylaxis among non-HIV immunocompromised patients and tried to define the type of immunocompromised patient that would benefit from PCP prophylaxis (Stern et al., 2014). Thirteen trials were included involving 1412 patients. These trials included 520 children with acute lymphoblastic leukemia; the remaining trials included adults with acute leukemia, solid organ transplantation or bone marrow transplant recipients. There was an 85% reduction in the occurrence of PCP in patients receiving prophylaxis with Trimethoprim/sulfamethoxazole (TMP/SMX) (RR of 0.15; 95% CI 0.04 to 0.62) compared to no treatment. Many international reference networks have published patient population-specific guidelines on when PCP prophylaxis is indicated. In this observational retrospective study, we investigate the proportion of patients admitted with a diagnosis of PCP who did not receive proper prophylaxis according to specific guidelines for HIV-positive, hematology and solid organ transplant patients and hospital-specific guidelines. (Maertens et al., 2016; Maschmeyer et al., 2016; White et al., 2017)

## Methods

### Study Design

We performed a retrospective multicenter cohort study in 3 large tertiary referral centers (AZ Sint-Jan Bruges, Belgium; University Hospital Ghent, Belgium and Erasmus University Medical Centre, Rotterdam, The Netherlands). A list of patients 18 years or older in whom a *P. jirovecii* real-time quantitative PCR (qPCR) was performed on a BAL sample between September 2012 and March 2018, was retrieved from the clinical microbiology laboratories of the three participating hospitals. Medical charts of all patients were reviewed for data extraction. The Institutional Review Boards of all sites approved the study.

### Definition

All three centers use an in-house qPCR to diagnose PCP. Diagnosing PCP usually depends on the histological or microscopic identification of ascus (cysts containing ascospores) and trophic forms in tissue, BAL or induced sputum using specific staining methods like Wright's-Giemsa or Grocott-Fomori stains. However, these methods are labor intensive and require specific expertise. Also, the fungal burden may vary substantially, in particular in HIV-uninfected patients which may limit the sensitivity of direct microscopy in these patients (Alanio et al., 2011). A conventional PCR can detect low concentrations *of* PJ DNA but low quantities of PJ DNA can be detected in asymptomatic patients as well. With the development of qPCR, it became possible to quantify the PJ load using the semi-quantitative Cycle Threshold (Ct) values (Sing et al., 2000; Alanio et al., 2011; Montesinos et al., 2017). Based on validation studies using microscopically confirmed cases of PCP, thresholds of Ct values have been proposed to differentiate between PCP and colonization with a ‘gray zone‘ in between. Unfortunately, most centers use an in house validated qPCR and therefore the cut-off values used to rule PCP in or out often differ between centers. The three clinical microbiology laboratories all used an in-house developed qPCR method that all targeted the mitochondrial rRNA (Desmet et al., 2009; Alanio et al., 2011; Steensels et al., 2015). Although the methods differed in primer-probe sequences and equipment for DNA extraction and amplification, their results are very similar as the reported semi-quantitative results by each of three laboratories for the same external quality control scheme were nearly identical and because the internal validation procedure in each laboratory resulted in similar cut off Ct-values to indicate probable PCP (Ct value ≤ 28.5-30.0) and probable *colonization* with PJ (Ct value > 34.0–35.0) (Table 1). These results demonstrate that the analytical variation between the three laboratories was very small and is neglectable compared with the biological variation and the variation caused by differences in sampling (e.g., place and volume of BAL). As a large range in Ct-values was considered an indeterminate result regarding the discrimination of PCP from PJ colonization, we applied the following case definitions for patients for all three hospitals:

- A case of *probable* PCP is defined as a patient with a positive qPCR result with a Ct value below the cut-off that was considered strongly suggestive of a PCP used in each of the centers (i.e., Ct 28.5– ≤30, Table 1). Furthermore, the patient should have received PCP therapy or the patient had died without PCP therapy with respiratory failure contributing to the death of the patient.
- A case of *possible* PCP was defined as a patient with positive qPCR results with a Ct value ≤34-35 and both of the following criteria: 1. A clinician's judgement that the illness was compatible with PCP based on the clinical and radiological findings AND 2. The patient died with respiratory failure contributing to the death of the patient or the patient received treatment with co-trimoxazole or another anti-PCP therapy (e.g., pentamidine IV, atovaquone) and survived.
- Patients with a positive qPCR result with a Ct value ≤34–35 that did not fulfill the definition of probable or possible PCP and patients with a positive qPCR result but with Ct values >34–35 were considered to be colonized with PJ.

**Table 1:** Interpretation of Ct value in each of the 3 centers regarding likelihood of PCP.

**Ct value interpretation**	**Erasmuc MC**	**AZ Sint-Jan**	**UZ Gent**
Strongly suggestive	≤29	≤28.5	≤30
Possible	>29 and ≤34	>28.5 and ≤34	>30 and ≤35
Unlikely	>34 and ≤40	>34 and ≤40	>35
Negative	>40	>40	>40

Patients with no sufficient clinical data provided in the medical chart were excluded. At the end of data collection all PCP cases were reviewed by AD, AS, and BR.

### Statistical Analysis

Statistical analysis included descriptive statistics including numbers (percentages) and medians (ranges) for categorical variables and continuous variables, respectively. Data were reported as percentages for categorical variables and as mean ± standard deviation (SD) or median with interquartile range (IQR) for continuous variables. Univariable statistical analysis was performed using independent *t*-test or Mann–Whitney *U*-test and Pearson's chi-square test. All the tests of significance were two-tailed and defined as *p* < 0.05 and analysis was performed with SPSS version 21 (IBM, Armonk, NY, USA). The following variables were registered for each patient: demographic parameters, underlying diseases, PCP prophylaxis (product, dose, duration), glucocorticoid use (dose, duration), symptoms (fever, dyspnea, nonproductive cough), duration hospital stay, ICU admission and duration, mechanical ventilation, mortality, PCP therapy, and prophylaxis indication following the guidelines (White et al., 2017).

### Prophylaxis

All patients were reviewed if they had received PCP prophylaxis and if prophylaxis was indicated according to the appropriate guidelines for prophylaxis and treatment of PCP. The following guidelines were used: Center of Disease Control and Prevention, the National Institutes of Health, and the HIV Medicine Association of the Infectious Diseases Society of America (Kaplan et al., 2009), European Conference on Infections in Leukemia (Maertens et al., 2016) and the American Society of Transplantation (Martin and Fishman, 2013; White et al., 2017).

The local guideline was provided by the Erasmus Medical Center and stated that prophylaxis should be given to the following patient groups as seen in Table S1.

## Results

**Figure 1:**
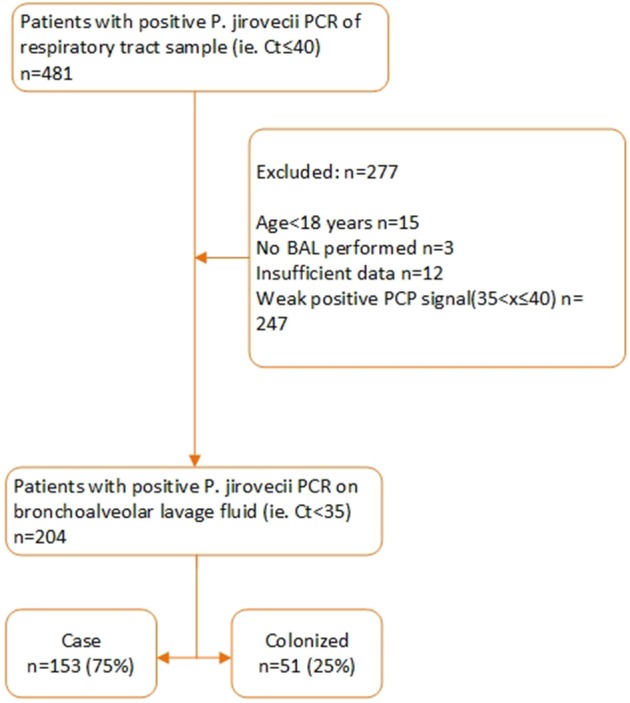
Overview of the inclusion process.

**Figure 2:**
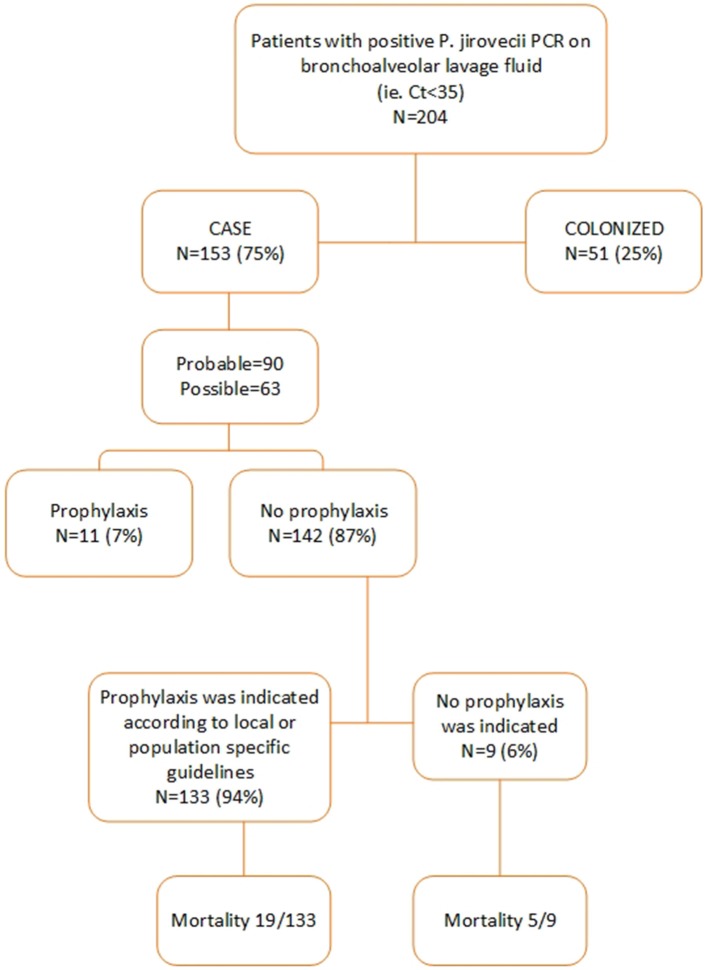
Overview of the (non-)use of prophylaxis.

Between September 1, 2012, and May 31, 2018, a respiratory tract specimen of 481 patients had a positive PJ qPCR test result. 277 of these patients were excluded for further analysis for the following reasons: age< 18 (*n* = 15), specimen tested was not a BAL (*n* = 3), insufficient clinical data (*n* = 12), Ct value >35 (*n* = 247). Two hundred and four patients were retained for further analysis of which 153 were defined as probable or possible PCP cases while 51 were considered colonized. Of these 153 patients, 90 had a probable and 63 a possible PCP. Eleven of these 153 patients (7%) received PCP prophylaxis according to guidelines while 142 (93%) did not. According to the patient-specific guidelines, 133 (87%) of the 153 patients had an indication for PCP prophylaxis (Figures 1, 2). In Table S2, the proportion of patients per patient group is shown that did not receive prophylaxis, although it was indicated.

**Figure 3:**
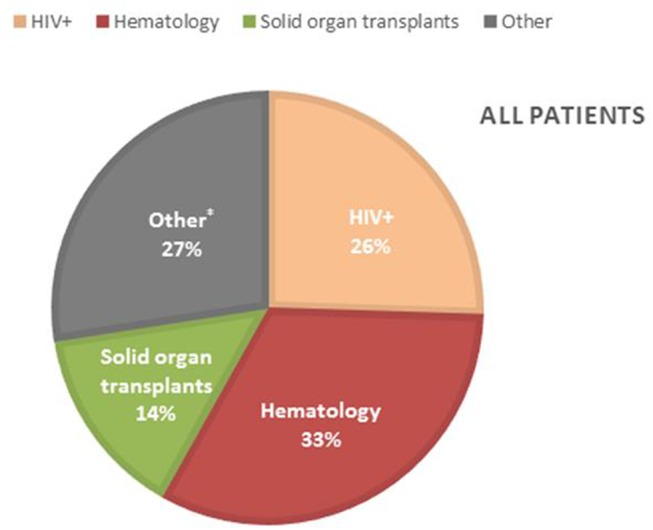
Incidence of PCP in the different PCP patient groups. *Other patients; lung diseases (11), patients with malignancy other than hematology (11), (metastatic) brain tumors (6), dermatology patients (4), auto-immune diseases (4), rheumatoid arthritis (2), hyperthyroidism (1), mental health patient (1), unknown (2).

Patients characteristics of all 153 probable and possible PCP cases can be found in Table S3. Mean age of all PCP patients was 57 years (±16 SD), 69.3% were males and 39 (25.5%) were HIV-positive. The non-HIV infected PCP patients consisted of 50 (32.7%) hematology patients, 22 (14.4%) solid organ transplantation recipients and 42 (27.4%) were patients with a mix of other underlying diseases for which immunosuppressive therapy had been initiated (Figure 3). A total of 82 (71.9%) patients had received corticosteroids preceding the diagnosis of PCP with a median duration of 30 (IQR [15–31]) days and a median dosage of 24.5 ([12.5–40]) mg/day prednisone. The main reason for corticosteroid administration was SOT, allogeneic stem cell transplant or metastatic disease of a solid organ cancer. Overall, 11 (7.2%) patients received PCP prophylaxis of whom 5 patients used trimethoprim/sulfamethoxazole (TMP/SMX) and the other 6 patients used atovaquone or pentamidine although the retrospective nature of the study did not allow for the evaluation of actual compliance with the prescribed prophylaxis. 133 (87%) patients of 153 cases did not receive prophylaxis as indicated according to the local or population-specific guidelines. The indication for prophylaxis was the use of corticosteroids in the majority (74/133) of the cases. The median overall hospital stay was 20 (IQR [11–35]) days and 14 (IQR [7–28]) days after the PCP diagnosis. ICU admission was required in 58 (37.9%) patients with a median duration of 10 (IQR [4–16]) days. Intubation and mechanical ventilation was required in 41 of the 153 (71%) with a median duration of 7 (IQR [4–16]) days. In total, 29 of the 153 patients with PCP (19%) died before hospital discharge. Adjunctive corticosteroid therapy was prescribed in 14 of the 29 (48.3%) patients that died. Mortality of patients with and without adjunctive corticosteroid therapy was similar at 13 and 16%, respectively.

A higher proportion of the HIV uninfected patients were admitted to the ICU and required mechanical ventilation (Table S4). Overall, 29 of the patients diagnosed with PCP died and 26 of them were HIV uninfected.

## Discussion

With the current study, we aimed to describe the epidemiology of PCP in patients admitted to three large tertiary care centers in a resource-rich setting. In particular, we focused on compliance with guidelines on the use of PCP prophylaxis preceding the PCP diagnosis. We classified patients into a probable or possible PCP, based on clinical and qPCR results. Of the 153 patients identified, 75% were immunocompromised due to an underlying disease other than HIV. This shift toward HIV-negative immunocompromised patients was described previously and is not surprising in the Netherlands and Belgium. Indeed, the WHO 90/90/90 goal regarding HIV management, defined as 90% of prevalent HIV infections being diagnosed, 90% of diagnosed cases on treatment and 90% of treated patients having an undetectable viral load, has already been achieved in both countries (Avino et al., 2016; White et al., 2017; van der Valk et al., 2019).

The key observation of our study was that 133 of the 153 patients (87%) diagnosed with PCP had not received prophylaxis while they had an indication for it according to patient-specific guidelines. However, the effectivity of prophylaxis is without a doubt. Indeed, a Cochrane meta-analysis demonstrated that prophylaxis is very effective with a relative risk for PCP of 0.15 when Trimethoprim/sulfamethoxazole (TMP/SMX) is used (Stern et al., 2014). We agree however that the severity of immunosuppression above which prophylaxis is required remains a matter of debate. While the importance of prophylaxis for patients with HIV not on cART and with a CD4 count below 200 cells/μL is without a doubt, the dose and duration of corticosteroid therapy above which the dose and duration of corticosteroid therapy above which the benefit to risk ratio of PCP prophylaxis becomes positive is not well-established. To generate these data, a large cohort of patients on corticosteroid therapy, while not on PCP prophylaxis should be followed over a long enough time to define the PCP incidence per 100 years of follow-up at a particular cumulative and daily corticosteroid dose. This is a challenging exercise which the current data, unfortunately, did not allow to do. Fortunately, Park JW et al. very recently described the results of a large retrospective study on the incidence of PCP in patients with rheumatic diseases on corticosteroid therapy (Park et al., 2018, 2019). They found an incidence of PCP of 2.4 per 100 person-years of follow-up for patients receiving a daily dose of >30 mg prednisone equivalent and 0.5 per 100 person-years in those on a dose of 15-30 mg per day. In the latter group, 4 of the 5 patients with PCP had to receive concomitant steroid pulse therapy on top of the daily dose. Therefore, when these pulse therapies are included in the calculation of the mean daily prednisone dose, 4 of the 5 patients with PCP could be considered to have received a dose >30 mg per day and the 5th patient had as an important additional risk factor an absolute lymphocyte count <200 cells/μL. Therefore, a 30 mg threshold above which PCP prophylaxis is indicated may be reasonable as long as steroid pulse therapy is considered a risk factor as well.

But even if a consensus can be reached on the dose above which the benefits of PCP prophylaxis outweigh the risk of prophylaxis, other barriers toward improved compliance with PCP prophylaxis remain to be tackled. Indeed, despite the fact that local PCP guidelines were in place also for non-SOT non-hematology patients on corticosteroids above the dose described in Table S1 at one of the 3 hospitals of our study, prophylaxis was not used in the majority of these PCP cases. Given the broad use of corticosteroids as immunosuppressive drug across a wide range of diseases covered by many medical specialties, it is challenging to make every clinician aware of when PCP prophylaxis is indicated. If digital medication orders are used, an automatic reminder on the use of PCP prophylaxis that pops up when corticosteroids are prescribed above a certain dose comparable to drug-drug interaction warnings may improve compliance with prophylaxis. But even then, overload of these warnings may lead to fatigue and eventually not lead to the desired effect. Therefore, awareness of the problem is needed. But even then, clinicians may remain reluctant regarding the use of prophylaxis due to the lack of conclusive evidence on when the benefit-risk ratio of PCP prophylaxis becomes positive.

Unfortunately, the onset of PCP in HIV-negative patients is often abrupt with respiratory failure at the time of diagnosis (Morris and Norris, 2012). The higher mortality (22.8%) that we observed in the HIV-negative patients compared with the HIV-infected (7.7%) and the longer hospital stay illustrated this.

Our study has its limitations. Given its retrospective nature, we were unable to assess the reasons for the non-use of prophylaxis by the treating physician. Also, the classification of cases into probable and possible PCP is somewhat arbitrary and the qPCR Ct values used in each of the hospitals differed slightly. However, we tried to use definitions that match clinical practice as much as possible. Also, a BAL procedure is often not standardized regarding the location inside the lung where sampling is performed nor the volume that is instilled and aspirated. As already discussed above, the second limitation of our study is the criteria used regarding PCP prophylaxis being defined as indicated for the HIV-uninfected, non-hematology, non-SOT patients. The guideline used at Erasmus MC regarding PCP prophylaxis in this very heterogeneous group of immunocompromised patients was based on expert opinion and limited retrospective observational data rather than conclusive evidence from prospective clinical trials. It states that prophylaxis is indicated when prednisone at a dose of >20 mg is given for >21 days. Given the recent results by Park JW et al. already discussed in detail above, a 30 mg threshold may be more appropriate as long as pulse therapy with corticosteroids is included in the calculation of the daily dose. Finally, although we feel that the 3 hospitals are representative of tertiary care hospitals in Belgium and the Netherlands, compliance with PCP prophylaxis guidelines may be better in other hospitals.

In summary, in our case series, the majority of patients diagnosed with PCP in recent years had not received PCP prophylaxis even though patient-specific guidelines recommending prophylaxis were available for most. PCP was mostly diagnosed in HIV-negative patients and mortality in this population was high. Awareness of the risk of corticosteroid-based immunosuppressive therapy should increase, in particular among sporadic prescribers. Automatic warning systems incorporated in electronic patient files and/or digital medication prescription systems could facilitate this and should be studied.

## Data Availability Statement

The datasets generated for this study are available on request to the corresponding author.

## Ethics Statement

The studies involving human participants were reviewed and approved by METC, Erasmus MC, Ethische Commissie, UZ Gent, and Belgium Ethische Commissie. Written informed consent for participation was not required for this study in accordance with the national legislation and the institutional requirements.

## Author Contributions

AS and BR designed the study. AD, AS, and SA coordinated the information technology and database. JH, MR, SV, JB and PD helped with data extraction. SA and AS collected the data. SA, AD, BR, and AS analyzed the data. AD, AS, and SA completed the statistical analysis. AD, AS, and BR wrote the first draft. All authors revised the manuscript.

## Conflict of Interest

The authors declare that the research was conducted in the absence of any commercial or financial relationships that could be construed as a potential conflict of interest.
