# Supplementary material for: Epidemiology of Pneumocystis jirovecii Pneumonia and (Non-)use of Prophylaxis
Source: Front Cell Infect Microbiol. 2020 May 15;10:224. doi: 10.3389/fcimb.2020.00224 (PMC7243458; doi:10.3389/fcimb.2020.00224)
Supplement: Supplementary file 1 [file Data_Sheet_1.docx]

| The following patient groups are eligible for PCP prophylaxis |
| --- |
| On top of the typical risk groups (HIV and CD4 <0.2*109/L, solid organ or allogeneic stem cell transplantation) other immune-compromised patients can be at risk for *Pneumocystis jiroveci* pneumonia (PCP).  The following patients are therefore eligible for PCP prophylaxis:   - Patients who receive> 20 mg prednisone equivalent per day for> 3 weeks. A high dose of pulse therapy (e.g. 500 mg methylprednisolone every 4 weeks) applies as well here. - Patients on treatment that results in T-cell depletion (e.g. anti-thymocyte globulin).   Furthermore, PCP prophylaxis should be restarted in patients after organ transplantation when anti-rejection treatment is administered.  The PCP prophylaxis is discontinued after the orally administered immunosuppressive treatment has been completely withdrawn. When administering high dose IV prednisone pulse therapy and / or ATG, the prophylaxis is preferably not stopped until 12 weeks after the last therapy. |
| Drug of choice for PCP prophylaxis |
| *First choice:*   - Cotrimoxazole 480 mg once daily. This is the most effective and cheapest.   *Alternatives:*   - Dapsone 100 mg once daily (not in patients with cotrimoxazole allergy). In non-Caucasian patients exclude G6PD deficiency before prescribing Dapsone. - Atovaquone solution 1500mg once daily to be taken during or immediately after a fat-containing meal. - Pentamidine nebulization: 300mg once daily every 4 weeks in a dedicated nebulizing room available in the hospital |

Table S1: Overview of patient groups where *Pneumocystis jirovecii* pneumonia (PCP) prophylaxis should be considered and overview of drug of choice for PCP prophylaxis following the Erasmus MC guidelines.

| cases | total *(N)* |  | prophylaxis (YES) | prophylaxis (NO) | INDiCATION^1^  yes no | |
| --- | --- | --- | --- | --- | --- | --- |
| HIV+ | 39 |  | 1 | 38 | 38 | 0 |
| HIV- | 114 |  | 10 | 104 | 84 | 17 |
| - HEMATOLOGY PATIENTS | 50 |  | 6 | 44 | 38 | 6 |
|  |  |  |  |  |  |  |
| - SOLID ORGAN TRANSPLANTATION | 22 |  | 4 | 18 | 18 | 0 |
| - OTHERS ^2^ | 42 |  | 0 | 42 | 28 | 11 |
| MISSING *(N)* |  |  |  |  | 3 |  |

Table S2: The use of prophylaxis among patient groups

^1^: Patients were supposed to receive PCP prophylaxis according to local and or international guidelines

| baseline characteristics | PCP cases |
| --- | --- |
| total | 153 |
| age (years) (sd) | 57(16) |
| sex(man), *n*(%) | 106(69.3) |
| hiv+, *N*(%) | 39(25.5) |
| HIV-, *N*(%) | 114(74.5) |
| hematology, *n* | 50(32.7) |
| solid organ transplantation, *n* | 22(14.4) |
| other patients*, *n* | 42(27.4) |
| corticosteroid use^1^, yes (%) | 82(71.9) |
| corticosteroid DURATION in DAYS | 30[15- 31] |
| median dose^1^ , (mg/day)[IQR] | 24.5[12.5-40.0] |
| prophylaxis use, (yes)(%) | 11(7.2) |
| type of prophylaxis |  |
| 1. Trimethoprim/sulfametoxazole, *N*(%) | 5(3.3) |
| 1. other, *N*(%) | 6(3.9) |
| prophylaxis indication^2^   - Corticosteroid USE - Other Immunosuppressive agents - Chemotherapy - HIV with CD4 <200 | 133(86.9)  74  8  13  38 |
| outcome |  |
| icu, (yes )(%) | 58(37.9) |
| MEDIAN icu duration, days [iqr] | 10[4-16] |
| mechanical ventilation (MV) (%) | 41(26.8) |
| median mv duration, days [IQR] | 7[4-16] |
| median hospital stay total, days [IQR] | 20[11-35] |
| median hospital stay after PCP diagnosis, days [IQR] | 14[7-28] |
| Hospital Mortality(%)   - ADJUNCTIVE THERAPY | 29(19)  14 |
| therapy, (yes)(%) | 153 |
| UNKNOWN MISSING, *N* | 3 |
| 1. Trimethoprim/sulfametoxazole, *N*(%) | 121(24.2) |
| 1. therapy+adjunctive corticosteroid, *N*(%) | 85(55.6) |
| 1. clindamycine +primaquine, *N*(%) | 22(13,7) |
| 1. atovaquon, *N* (%) | 2(1,3) |
| 1. dapson+trimetroprim, *N*(%) | 4(2,6) |
| 1. pentamidine, *N*(%) | 1(0,7) |
| MEDIAN therapy duration, days [IQR] | 20[13-21] |

Table S3: Baseline characteristics for the PCP cases

^1^: corticosteroid use and dose only for the HIV – patients.

^2^: prophylaxis as indicated by ref. 8, 12, 13 or according to Erasmus MC guidelines (table S1 and S2).

^*^: Other patients; lung diseases (11), patients with malignancy other than hematology (11), (metastatic) brain tumors (6), dermatology patients (4), auto-immune diseases (4), rheumatoid arthritis (2), hyperthyroidism (1), mental health patient (1), unknown (2)

| All cases (153) | HIV-(114) | HIV +(39) |
| --- | --- | --- |
| DEAD *(yes)* | 26 (22.8%) | 3(7.7%) |
| Mortality, days AFTER diagnosis | 19[16-22] | 13.5[7-24] |
| ICU *(yes)* | 50(43.9%) | 8(20.5%) |
| MV *(yes)* | 34(29.8%) | 7(17.1%) |
| Adjunctive corticosteroid as treatment (*n)* | 61(53.5%) | 24(61.5%) |

Table S4: Survival status between non-HIV and HIV-infected patients.
